# Tumor Location and Referral Sources as Determinants of Late-Stage Oral Cancer Diagnosis in a Portuguese Cohort

**DOI:** 10.7759/cureus.95982

**Published:** 2025-11-03

**Authors:** José Cunha Coutinho, Leonor Cruz e Silva, Ricardo São João, Gonçalo Cunha Coutinho, Tiago Domingues, Miguel Araújo Nobre, Cecília Caldas, Francisco Salvado

**Affiliations:** 1 Stomatology, Centro Hospitalar Lisboa Ocidental, Lisbon, PRT; 2 Stomatology University Clinic, Santa Maria University Hospital Centre, Lisbon, PRT; 3 Computer Science and Quantitative Methods, School of Management and Technology, Polytechnic Institute of Santarém, Lisbon, PRT; 4 Centre of Statistics and Its Applications, Faculty of Sciences, Universidade de Lisboa, Lisbon, PRT; 5 Stomatology University Clinic, Faculdade de Medicina da Universidade de Lisboa, Lisbon, PRT

**Keywords:** early diagnosis, gingival squamous cell carcinoma, oral cancer detection, oral cancer referral, oral squamous cell carcinoma, risk factors, tumor characteristics

## Abstract

Background: Most cases of oral cancer at the time of diagnosis are already locally advanced, which is associated with a worse prognosis. This study aimed (1) to identify tumor characteristics and risk factors associated with late-stage oral cancer diagnosis in the Portuguese population and (2) to develop population- and provider-oriented early detection strategies, particularly focused on recognizing potentially malignant disorders, to enhance timely diagnosis and improve patient outcomes.

Methods: A retrospective study of 151 patients with oral cancer treated in a Portuguese tertiary hospital between January 2015 and April 2023 was conducted. Information regarding sociodemographic variables, habits, tumor location and form of presentation, referral sources, and stage of the disease at initial diagnosis was analyzed.

Results: The majority of patients were male, and the mean age at diagnosis was 65 years. Most were diagnosed at stage IV (51.7%, n = 75), and most of these were referred by the emergency department (46.7%, n = 35). Family physicians made the earliest referrals. Patients referred by dentists were diagnosed at more advanced stages of disease by comparison. The anatomical sites with the highest percentage of locally advanced disease were the lower gingiva, retromolar trigone, and upper gingiva.

Conclusions: Late-stage oral squamous cell carcinoma was the most common diagnosis, particularly for lesions in the gingiva and retromolar trigone, and was more frequent among cases referred through emergency departments, reflecting gaps in early detection. Targeted surveillance, improved professional training, and public awareness are essential to promote earlier diagnosis.

## Introduction

Oral squamous cell carcinoma (OSCC) represents more than 90% of oral cavity cancer cases. Classic risk factors include tobacco smoking and alcohol consumption. In many cases, it develops through malignant transformation of oral potentially malignant lesions, namely leukoplakia, erythroplakia, and leukoerythroplakia [1].

OSCC may present locally as a persistent ulcerated lesion, an erythematous area, or leukoplakia of the oral mucosa, and may also develop regionally due to metastasis, usually as a single or multiple cervical lymphadenopathies. It can manifest symptomatically, with pain, bleeding, and dysphagia, or remain asymptomatic and may be detected incidentally during medical or dental examination.

Current knowledge of carcinogenesis suggests that the longer the tumor exists, the greater its size and the likelihood of regional spread [2]. The rate of progression, however, is difficult to predict due to variations in the growth rate and aggressiveness of individual tumors. Factors affecting prognosis and patient survival include the site of the lesion, the size of the lesion at diagnosis, the degree of histopathological differentiation, regional lymph node involvement, and the presence of distant metastases [3].

Universal screening strategies are not currently recommended globally [4]. Diagnosis often follows the presentation of suspicious symptoms and signs to the attending general practitioner or general dentist. Despite this, data show that at the time of diagnosis, most cases are already in a locally advanced stage [5]. Clinical stage at diagnosis is recognized as an important prognostic marker, and five-year mortality rates vary significantly with it [6]. The five-year survival rate is greater than 80% in patients with localized disease at diagnosis, as opposed to rates of less than 30% in patients diagnosed with advanced-stage disease [7].

The longer the diagnostic delay, the more advanced the staging [8]. Radical surgical treatment is considered the gold standard in OSCC; however, in locally advanced cases, it entails great morbidity with disfigurement, impaired communication and feeding, social isolation, and sometimes death [9].

Several studies reveal that more than half of OSCC cases are diagnosed at advanced stages, despite the oral cavity being easily accessible for inspection [10]. Despite recent therapeutic advances, the overall survival rate has not improved in recent decades. As such, the focus has been directed toward the importance of early diagnosis as a potential factor in increasing survival in this neoplasm [11].

Accordingly, the present study was designed with two main objectives: first, to identify tumor characteristics and risk factors associated with late-stage oral cancer diagnosis in the Portuguese population, and second, to propose early detection strategies directed toward both the general population and healthcare providers, emphasizing the recognition and timely management of potentially malignant disorders as a means to improve diagnostic timing and patient survival.

## Materials and methods

A retrospective study was conducted on patients diagnosed with OSCC and followed at the Stomatology Department of a Portuguese tertiary care university hospital between January 2015 and April 2023. The study was approved by the Institutional Ethics Committee and conducted in accordance with the Declaration of Helsinki. All patient data were anonymized before analysis to ensure confidentiality.

All consecutive cases with histopathological confirmation of OSCC were included. The exclusion criteria were the absence of sociodemographic data, tumor location and clinical presentation information, a histopathology report, or missing staging information. When other variables, such as smoking or alcohol habits or referral source, were unavailable, patients were retained in the dataset, and analyses were performed using the available data for each variable.

The dependent variable consisted of disease stage at diagnosis, classified according to the American Joint Committee on Cancer (AJCC, 8th edition) prognostic stratification groups.

The independent variables included sociodemographic data such as sex and age (measured in years) and patients’ current or past habits of smoking (quantified in pack-year units (PY)) and alcohol consumption. Tumor characteristics included location in the oral mucosa and form of presentation (homogeneous or heterogeneous aspect, color, endophytic or exophytic growth, and size). The origin of referral was also recorded, whether from the hospital emergency department, health center (family medicine physician), dental practice, stomatology consultation, or other medical specialties.

Descriptive and inferential statistical analyses were performed. For continuous variables, the mean ± standard deviation or median and respective interquartile range were presented, depending on the underlying data distribution. The normality of distributions was assessed using the Kolmogorov-Smirnov test with Lilliefors correction. For categorical variables, the results were presented as absolute frequencies (n) and percentages (%).

For comparison of continuous variables between two independent samples, the independent-samples t-test or Mann-Whitney U test was used, as appropriate. For comparisons involving more than two independent samples, the Kruskal-Wallis test was applied. For categorical variables, the chi-square test was used; when its assumptions were not met, the Fisher-Freeman-Halton exact test was applied. The chi-square test was used to assess associations between tumor location, stage, and degree of differentiation. Cochran’s criteria were verified for each test; when these criteria were violated, Fisher’s exact test or its extension (the Fisher-Freeman-Halton test) was applied. For each statistical test performed, when applicable, the test statistic (ET), degrees of freedom (df), and p-value were reported in parentheses. The significance level was set at 5%, and all statistical analyses and graphical representations were performed using the R programming language (version 4.3.3, R Foundation for Statistical Computing, Vienna, Austria).

## Results

**Figure 1:**
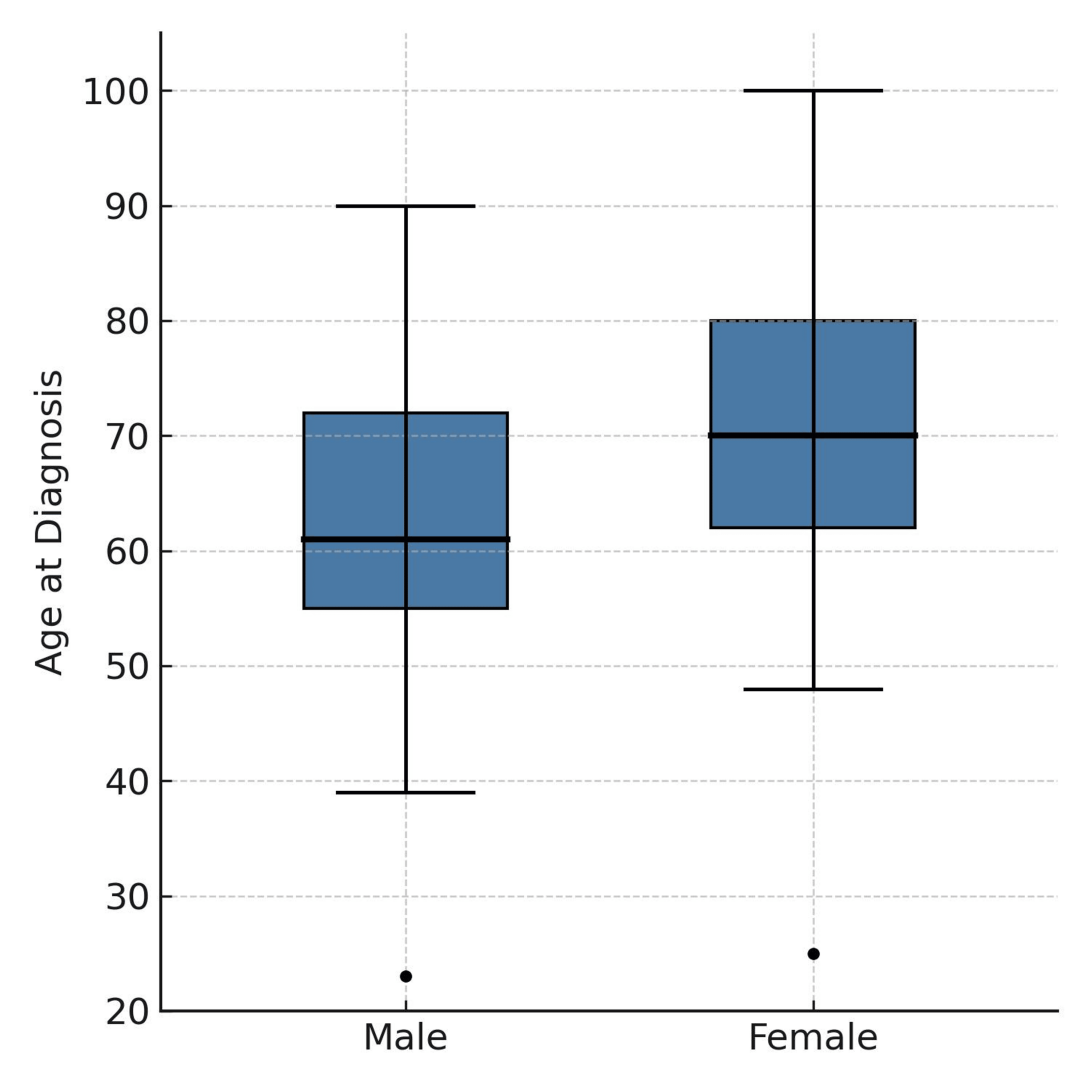
Age distribution at diagnosis by sex

The sample consisted of 147 patients diagnosed with squamous cell carcinoma (SCC) of the oral cavity after excluding four patients due to incomplete staging information. The majority were male (n = 87; 59%), and the mean age at diagnosis was 65 ± 13 years (range, 24-100 years). On average, older individuals were observed among females (t-test: male, 62.38 ± 13.13 vs. female, 69.25 ± 13.73; ET = -3.10; df = 148; p = 0.002) (Figure 1).

We registered 87 (59%) individuals with a history of smoking, and of these, 63 (72%) were active smokers, with an average consumption of 40 pack-year units (PY). There was a history of alcohol abuse in 74 (51%) subjects, and among these, 66 (89%) were active drinkers. The percentage of patients with current or past habits of both alcohol and tobacco use was 45.6% (n = 67). The percentage of patients with a history of cancer was 18.8% (n = 27), and about 6% (n = 9) had immunosuppressive conditions.

Most lesions had an ulcerated surface (82.2%, n = 97) and exophytic growth (56.0%, n = 51). The most frequent presentation with color change of the oral mucosa was red-and-white lesions (41.7%, n = 15).

The most frequently diagnosed type of OSCC by pathology report was conventional (93.3%, n = 139) with histological differentiation grade 1 (65.0%, n = 93).

Considering the prognostic stratification groups, 29 (20%) patients were diagnosed in early stages (stage 0 or stage I), 25 (17%) patients were diagnosed in stage II, 16 (11%) patients were diagnosed in stage III, and most patients (51.7%, n = 75) were diagnosed in advanced stage (stage IV) (Table 1).

**Table 1:** Patient characteristics

Variable	Value
Smoking status, n(%)	
Never	60 (40.8)
Active	63 (42.9)
Ex-smoker	24 (16.3)
Alcohol status, n(%)	
Never	72 (49.3)
Active	66 (45.2)
Ex-consumer	8 (5.5)
Immunosuppressive conditions, n(%)	
HIV	4 (44.4)
Primary immunodeficiency	1 (11.1)
Bone marrow transplant	2 (22.2)
Solid organ transplantation	2 (22.2)
Oncological background, n(%)	27 (18.8)
Referencing, n(%)	
Dentist	18 (12.3)
Stomatology consultation	12 (8.2)
Emergency stomatology	45 (30.8)
Family doctor	51 (34.9)
Other	20 (13.7)
Color, n(%)	
White	12 (33.3)
Red	9 (25.0)
White and red	15 (41.7)
Growth, n(%)	
Endophytic	40 (43.9)
Exophytic	51 (56.0)
Surface of the lesion, n(%)	
Exophytic	2 (1.7)
Irregular	9 (7.6)
Regular	6 (5.1)
Ulcerated	97 (82.2)
Warty	4 (3.4)
Type of CPC, n(%)	
Basaloid	2 (1.3)
Conventional	139 (93.3)
Desmoplastic	1 (0.7)
Papillary	1 (0.7)
Sarcomatoid	3 (2.0)
Verrucous	3 (2.0)
Grade of differentiation, n(%)	
1	93 (65.0)
2	44 (30.8)
3	6 (4.2)
Stage of disease, n(%)	
0	1 (0.7)
I	28 (19.3)
II	25 (17.2)
III	16 (11.0)
IV	75 (51.7)

Most patients were referred for observation and diagnostic evaluation in our department by their family doctor (35.2%, n = 50), followed by the patient’s own initiative to use the Stomatology Emergency Department (ER) of our tertiary hospital center (31.7%, n = 45). Other referral sources included other medical specialties (n = 19), dentists (n = 17), and nonurgent consultation at our department (n = 11).

**Figure 2:**
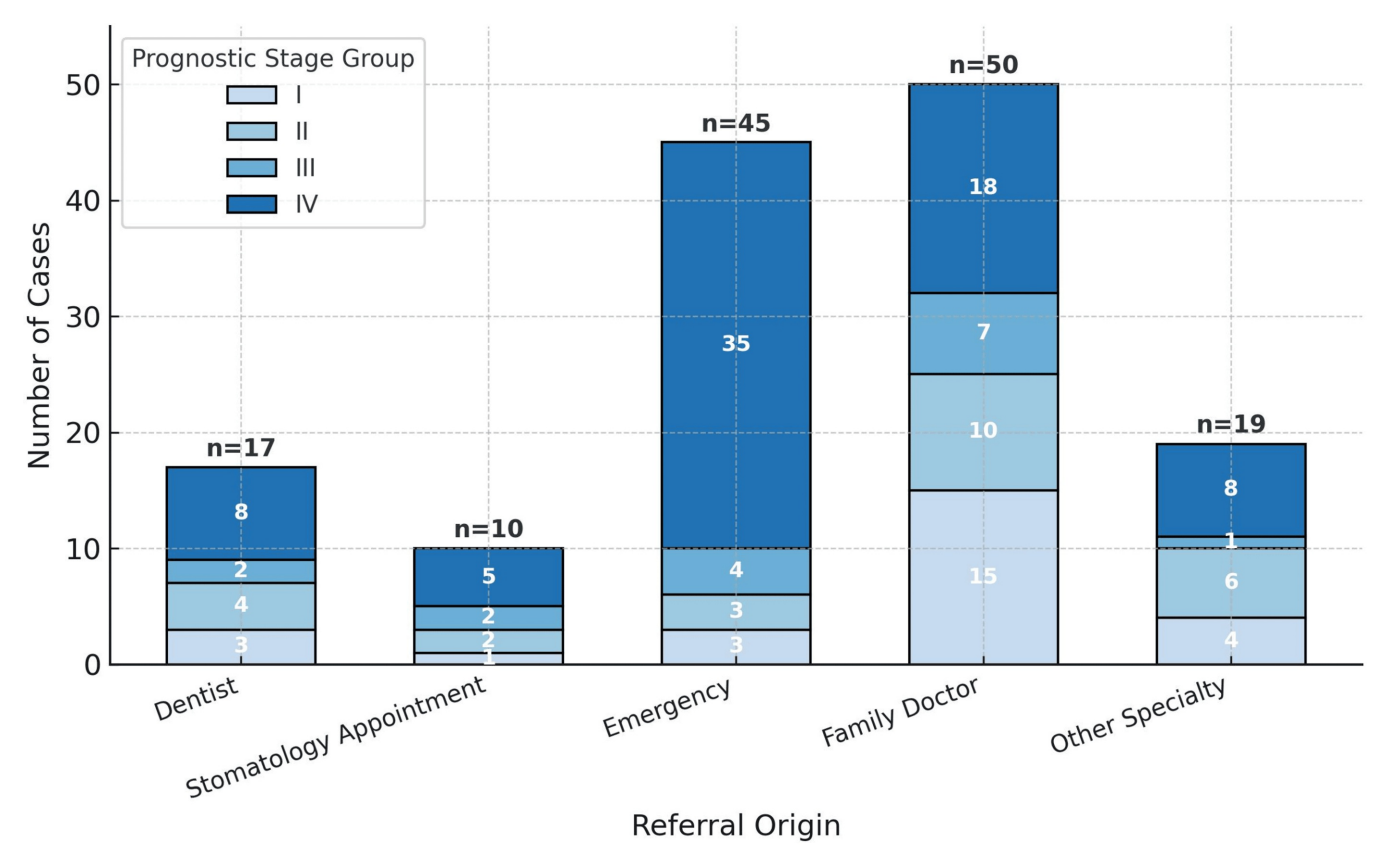
The number of cases versus referral origin versus AJCC prognostic stage group AJCC:  American Joint Committee on Cancer.

It was found that most cases of stage IV were referred by the emergency department (47.9%; Fisher’s exact test: p = 0.003). Conversely, family doctors made the earliest referrals, with 15 (30%) patients referred by a family doctor having stage I disease at initial diagnosis (stage I, 30% (n = 15); stage II, 20% (n = 10); stage III, 14% (n = 7); stage IV, 36% (n = 18)). Patients referred by their attending dentists were diagnosed at more advanced stages of disease (stage I, 17.6% (n = 3); stage II, 23.5% (n = 4); stage III, 11.7% (n = 2); stage IV, 47% (n = 8)) (Figure 2).

**Figure 3:**
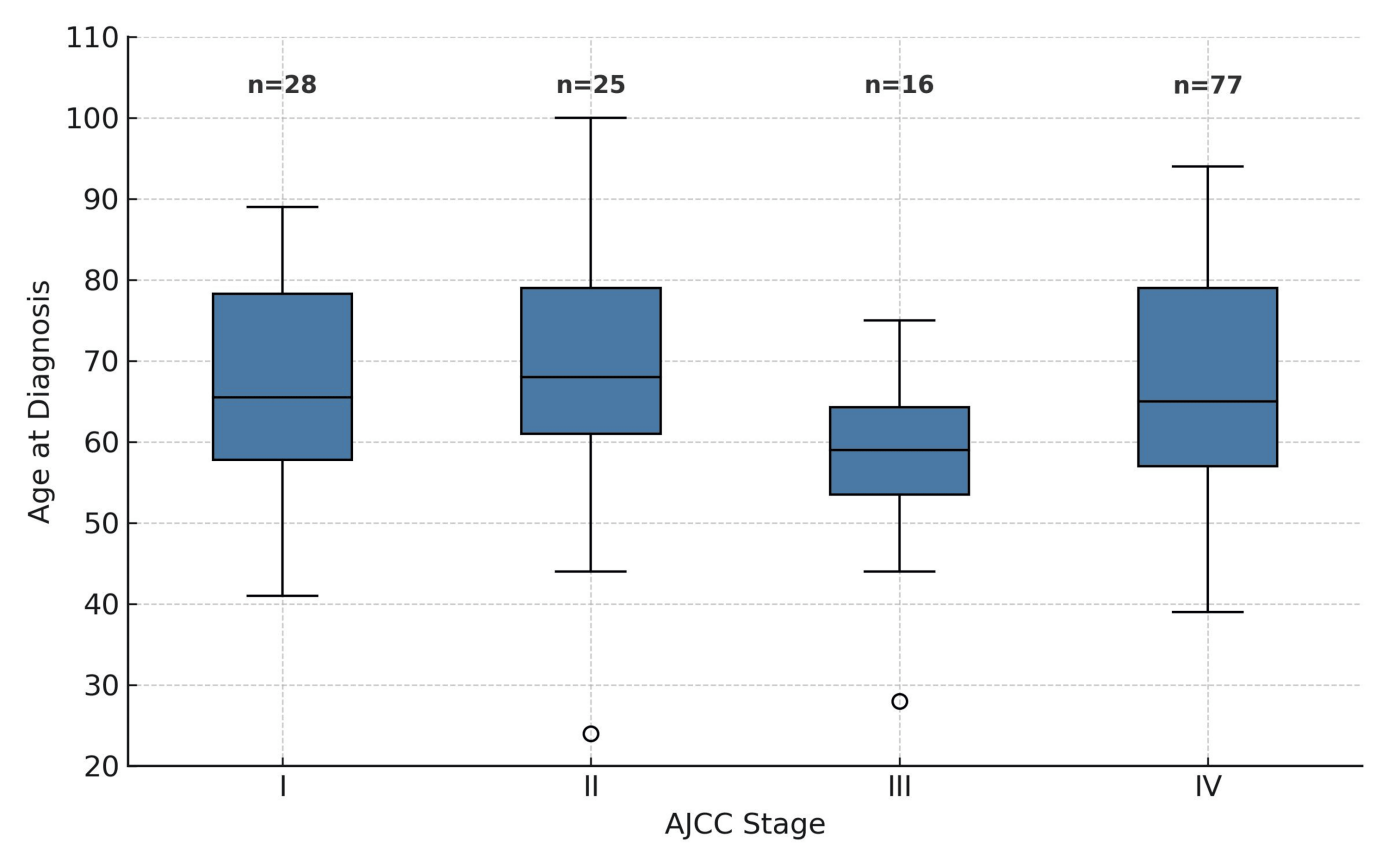
Age at diagnosis versus AJCC prognostic stage groups AJCC: American Joint Committee on Cancer.

There was no association between patient sex and disease stage at initial diagnosis (Fisher’s exact test: p = 0.94). In the comparison between disease stage and patient age, no significant differences were observed (median values: stage I, 65.5 (20.5) years; stage II, 68 (18) years; stage III, 59 (10.8) years; stage IV, 65 (20.8) years; Kruskal-Wallis test, p = 0.11) (Figure 3). There was no association between a history of neurological disease and disease stage (Fisher’s exact test: p = 0.98), nor between disease stage and smoking or alcohol habits (Fisher’s exact test: p = 0.15 and p = 0.96, respectively).

There were 39 lesions with endophytic growth and 50 lesions with exophytic growth. Of the tumors diagnosed as cT1, about 62% (n = 8) had an endophytic presentation. Of the locally advanced tumors (cT3 or cT4, n = 57), about 63% (n = 36) had an exophytic presentation. For tumors staged as cT2, the growth type showed equal distribution between endophytic and exophytic forms. Despite this pattern, no significant association was identified between the cT and growth variables (Fisher’s exact test; p = 0.135).

The locations with the highest percentage of locally advanced cases (cT3 and cT4) were the lower gingiva (95.2%, n = 20), the retromolar trigone (89.5%, n = 17), and the upper gingiva (81.8%, n = 9). All tumors of the upper lip (n = 2) and upper vestibule (n = 3) corresponded to locally advanced cases. Of the sites with a lower percentage of locally advanced cases and therefore associated with earlier diagnosis, the lower lip stood out (15.4%, n = 2), clearly distant from the others, which were the lateral border of the tongue (51.7%, n = 31), buccal mucosa (57.9%, n = 11), and ventral tongue (60.7%, n = 17). An association was found between tumor location and disease stage (cT) (Fisher-Freeman-Halton test extension; p < 0.001).

**Figure 4:**
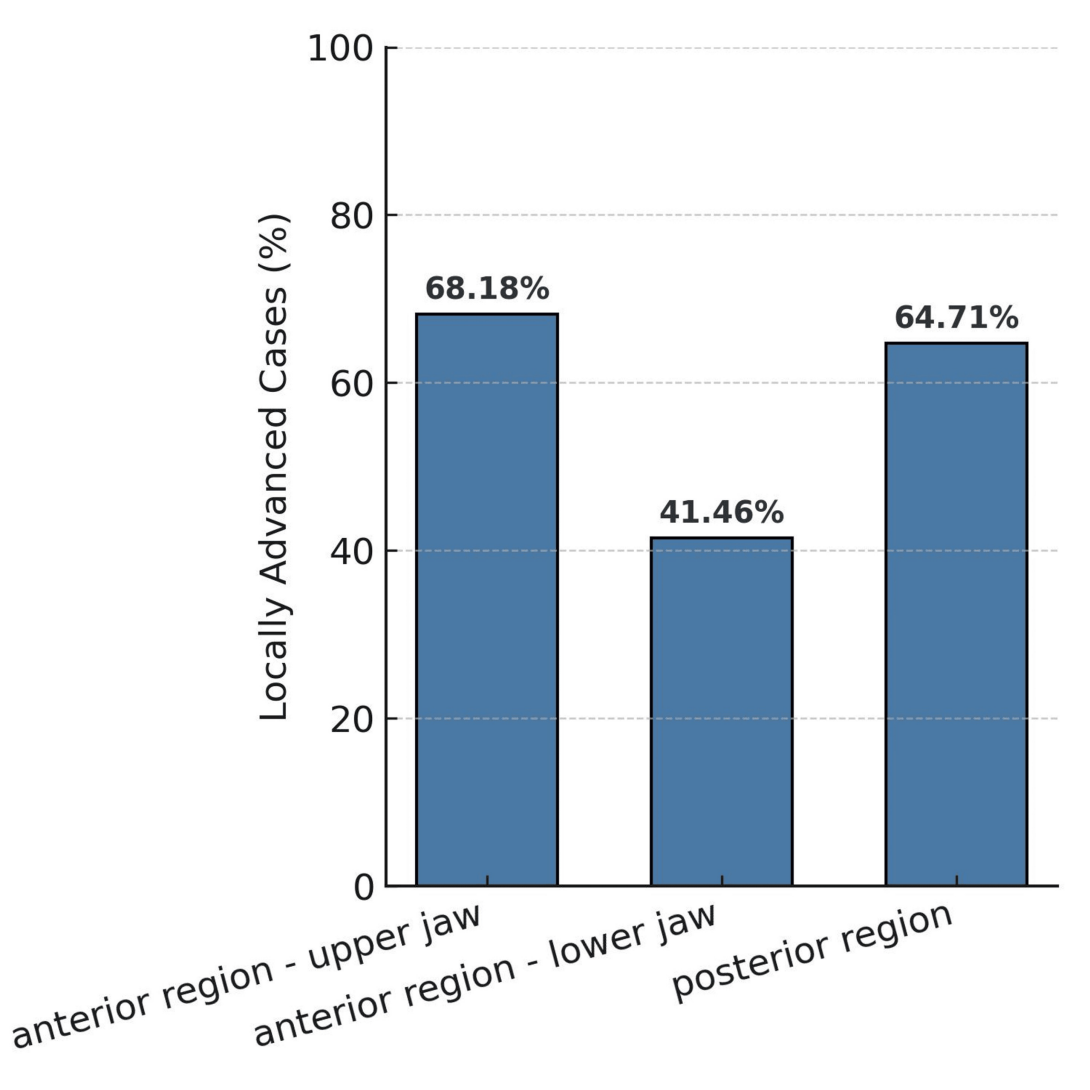
Percentage of locally advanced cases by region

Because some tumor sites had low case numbers, they were grouped into three clinically relevant anatomical regions: anterior maxilla (including upper lip, maxillary gingiva and alveolar ridge, upper vestibule, buccal mucosa, and hard palate), anterior mandible (including lower lip, floor of the mouth, mandibular gingiva and alveolar ridge, lower vestibule, and tongue tip and body), and posterior regions of the jaws (including the retromolar trigone, soft palate, and base of the tongue) (Figure 4).

The distribution of cervical metastasis varied significantly across these regions (chi-square test for proportions; p < 0.001).

No association was found between histological differentiation and disease stage at diagnosis (Fisher-Freeman-Halton test; p = 0.341).

## Discussion

In our cohort, we did not identify epidemiological characteristics significantly associated with the late diagnosis of oral cancer. In contrast to our study, Jafari et al. [12] pointed to more advanced-stage diagnoses in men. The same authors noted that men took longer from symptom onset to referral to primary health care (PHC); however, they observed no relationship between diagnostic delay and diagnosis at more advanced stages. Diagnoses in advanced stages of disease were also not related to smoking or alcohol habits, neurological disease, or to the endophytic or exophytic growth pattern. In summary, we found that initial diagnoses at advanced stages were significantly associated with tumor location, namely the gingiva and retromolar trigone, and with the origin of referral. We now address each of these aspects in more detail.

Similar to the present study, Seoane-Romero et al. [13] found an association between delayed diagnosis and tumor location in the retromolar trigone and gingiva. However, they also reported this association with tumors of the oral floor. Unlike the present investigation, they observed an association between less differentiated tumors and later diagnosis.

Both the gingival mucosa and the mucosa of the retromolar trigone are adherent keratinized mucosa. It is possible that tumors in this type of mucosa are more aggressive, as some authors have noted, particularly for tumors of the upper gingiva [14]. Another theory to consider is that because the adhered mucosa is close to the bone, even small tumors quickly reach and invade the cortical bone and are thus staged as locally advanced. This is particularly relevant for tumors of the upper and lower gingiva, which rapidly reach the alveolus and invade through the lamina dura, a thinner and more discontinuous compact bone, especially when patients also have periodontal disease [15]. This difference could explain why the same is not true for tumors of the adherent keratinized mucosa of the hard palate.

Another reason retromolar trigone tumors are often diagnosed late is their location in the posterior region of the oral cavity. These lesions are more difficult to observe, and when they are asymptomatic, they can take a long time to be identified by either the patient or the oral health professional. Furthermore, posterior and caudally located tumors of the oral cavity, such as those of the retromolar trigone, are also associated with more frequent nodal metastasis at initial diagnosis [16].

The late diagnosis of tumors of the gingiva may also be due to confusion with periodontal disease and benign gingival lesions [17,18]. These situations can delay diagnosis, leading to underestimation of the lesion and multiple unsuccessful dental treatments before neoplasia is suspected. Onizawa et al. [19] found that patients who first consulted dentists before diagnosis required, on average, more visits before being referred, and 23% underwent additional dental procedures.

The fact that tumors of the lower lip are the most likely to be diagnosed early is due to several factors: first, these lesions are easily identifiable on facial examination; second, many develop from actinic cheilitis, which is often already under surveillance. Another explanation may be that these tumors represent a form of SCC similar to cutaneous SCC, which is associated with sun exposure and tends to exhibit less aggressive behavior than SCC of the oral mucosa [20].

An additional factor related to later diagnosis of oral cancer was the source of referral. Unlike other cancers, OSCC can be diagnosed by two professional groups, physicians and dentists, and in different settings. Because of these differences, the referral pathway and subsequent patient visits can vary considerably. Previous studies have demonstrated variability in the professionals most frequently consulted prior to an oral cancer diagnosis. All studies except one, conducted by Onizawa et al. [19] in Japan, showed that patients more often visited a family doctor than a dentist [21-25]. Likewise, in our study, we found that most referrals from primary health care were made by family doctors. The cases associated with earlier diagnosis, as expected, originated from family doctors, who represent a first-line resource for patients presenting with complaints related to suspicious oral cavity lesions. Interestingly, the cases referred by dentists were diagnosed later compared to those referred by family doctors, even though dentists are also first-line professionals in diagnosing oral cancer. Two previous studies, one conducted in the UK and another in Brazil, sought to verify the relationship between the stage of disease at diagnosis and the origin of referral-physician or dentist. Contrary to the results of our investigation, in both studies, there were no significant differences [23,26]. Referral of cases in more advanced stages by dentists in our research may reflect a lower degree of suspicion regarding oral mucosal lesions, with a tendency to devalue or confuse them with other benign entities. It may also result from delayed referral by dentists, with additional visits and investigations preceding referral to a specialized center. Most dentist referrals in our study originated from private dental practices, which are less accustomed to managing malignant disease. Despite this, Tromp et al. [27] were unable to demonstrate an association between delays in referral and disease stage at diagnosis.

Most advanced-stage diagnoses occurred in cases presenting to the emergency department, as observed and reported in other studies [28]. Patient awareness plays an important role in reducing the time between the appearance of the first signs and symptoms and referral to primary health care. Reducing this lag time has the potential to decrease the total time from cancer onset to diagnosis and subsequent treatment. Strategies to achieve early diagnosis and treatment can substantially improve survival rates while reducing morbidity and overall health-care costs. These strategies should address all factors involved in diagnosis and treatment, namely, patient awareness, optimal pathways to primary and definitive diagnosis, therapeutic pathways, and recommended time intervals for each step from the first symptom to final treatment [29].

The present study has several limitations, including its retrospective, single-center design, which limits control over bias and confounding factors and restricts external validity; therefore, the findings should be interpreted with caution. Potential sources of bias include selection bias inherent to retrospective sampling, referral bias reflecting regional rather than national practices, and possible information bias due to variability in clinical documentation. Although data completeness was high, some variables were missing or inconsistently recorded, which may have affected analytical precision. Unmeasured confounders such as socioeconomic status, health literacy, and access to care could also have influenced diagnostic timing. Interobserver variability in staging and clinical assessment represents another potential source of error. Despite these limitations, the study benefits from strong internal validity and contributes to understanding the mechanisms underlying diagnostic delay within a defined population.

The proposed early detection strategies should be regarded as hypothesis-generating rather than causal and may guide future prospective, multicenter studies to validate and expand upon these findings. Future research should investigate diagnostic delays associated with suspicious gingival lesions and the clinicopathological factors related to malignancy. Furthermore, studies should assess whether small gingival SCCs (<2 cm) with bone invasion but no cervical metastasis (cT4aN0) exhibit different mortality outcomes compared with larger (>2 cm) nonkeratinized mucosal lesions without neck metastasis (cT2/3N0), despite representing a more advanced disease stage.

## Conclusions

This study highlights that late-stage diagnosis of OSCC remains alarmingly common, with tumor location and referral source appearing as important associated factors. Lesions of the gingiva and retromolar trigone are frequently misinterpreted as benign conditions and therefore more susceptible to diagnostic delay. The higher proportion of advanced-stage cases referred through emergency departments further suggests potential shortcomings in early detection within primary and dental care settings.

These findings point to the relevance of strengthening surveillance and educational initiatives aimed at earlier recognition of suspicious oral lesions, particularly among high-risk populations and healthcare providers. The proposed strategies should be viewed as exploratory and hypothesis-generating rather than causal, providing a basis for future prospective studies to validate and expand these observations.
